# Editorial: Interdisciplinary approaches to policing and mental health crisis

**DOI:** 10.3389/fpsyg.2026.1831322

**Published:** 2026-05-26

**Authors:** Mario S. Staller, Swen Koerner, Benni Zaiser

**Affiliations:** 1 Department of Police, University for Police and Public Administration North-Rhine Westfalia, Cologne, Germany; 2 Department of Training Pedagogy and Martial Research, German Sport University Cologne, Cologne, Germany; 3 Independent Researcher, Aurora, ON, Canada

**Keywords:** behavioral emergencies, co-response models, de-escalation, officer safety, professional reflexivity, trauma-informed communication, mental health crisis

Police interactions with people experiencing mental health or behavioral crises constitute a complex field in which public and officer safety, health needs, and institutional mandates collide under conditions of uncertainty and risk (Wittmann et al., 2020). In many jurisdictions around the world, police officers remain the default responders to behavioral health emergencies, even when the core problem is clinical, social, or relational. These encounters may involve acute distress, trauma histories, intoxication and substance use, psychiatric disorders, intellectual and developmental disabilities, and other neurodivergent presentations, as well as social marginalization, including homelessness, often with more than one factor present in combination. Mental health-related police encounters often unfold in public spaces with multiple audiences and constraints that shape what can be done, how quickly it has to happen, and what can and should be avoided from happening. Navigating these situations requires professional judgment and decision-making across competing knowledge bases and divergent “lenses” of observation, along with underlying knowledge, skills, and abilities (Bennell et al., 2022; Staller et al., 2022). These differences in expectations and meaning-making structure the interaction itself and can either enable de-escalation or amplify escalation dynamics.

Over the last decades, the field has made substantial progress in defining de-escalation principles, Crisis Intervention Team (CIT) approaches, and interagency collaboration models (see, e.g., Baltazar and Bang, 2025; Kamin et al., 2022; Lith et al., 2025; Zaiser et al., 2023). Yet tragic incidents around the world continue to indicate that further improvement is necessary, both in the availability of evidence-informed response systems and in the capacity for reflexive practice. Ensuring that people experiencing crisis are met with a minimally invasive footprint, harm reduction, and trauma-informed communication depends on integrating insights across disciplines, strengthening shared mental models, and cultivating professional reflexivity as a practical resource in moments where action and restraint must be calibrated in real time.

The articles collected in this Research Topic address these challenges and, in doing so, share a common recognition: Improving outcomes in mental health-related police encounters requires more than incremental refinements to training or policy. Rather, it requires a reframing of how crisis situations are conceptualized, how competencies are defined and assessed, and how responsibility for crisis response is distributed across systems. By drawing on perspectives from psychology, policing studies, behavioral science, occupational health, and social systems theory, the contributions collectively illuminate both the limits of traditional police-centric models and the potential of interdisciplinary approaches to reshape practice:

- Schade and Thielgen synthesize policing scholarship into a broader intervention model that clarifies how police actions unfold across phases, constraints, and intended effects. By mapping intervention logics, situational demands, and outcome pathways, this narrative review offers a scaffold that connects training, policy, and evaluation.
- Koerner et al. provide a systems-theoretical lens on how understanding is produced in crisis encounters. By distinguishing first-order understanding (making sense of the situation) from second-order understanding (understanding how one's understanding is formed and constrained), the article clarifies why misalignment can persist even when all actors aim for resolution. The analysis offers a conceptual bridge between de-escalation as a practical goal and reflexivity as a structural competency in police–citizen crisis interactions.
- Staller et al. re-position officer safety as more than a set of tactical prescriptions. The article argues for a reflexive concept (officer safety 2.0) that treats “control” itself as an object of professional observation and calibration, especially in encounters where escalation risk is co-produced by interaction dynamics.
- Lavoie et al. address a central bottleneck in the field: How to operationalize and measure de-escalation competencies in a way that is meaningful across disciplines.
- Watson, Jackson et al. focus on the practical conditions of producing reliable evidence in CIT research. By outlining the development and implementation of standardized scenarios, the article strengthens methodological foundations for training evaluation and cross-study comparability.
- Watson, McNally et al. widen the frame from police competencies to the design of crisis response systems. The article discusses the rationale for and requirements of developing alternative or complementary workforces in community behavioral health crisis response.
- Zaiser et al. offer a structured model that integrates assessment and response options for mental health calls. The iBEAR approach explicitly addresses heterogeneity—recognizing that “mental health crisis” is not a singular category and that response strategies should be differentiated accordingly.
- Marlatte et al. add an empirical layer to the psychophysiology of crisis work. By examining how physiological stress relates to cognitive performance during simulated encounters and to memory afterward, the article highlights mechanisms that matter for training transfer, decision quality, and post-incident reporting.
- Easton et al. foreground the cumulative psychological burden that investigative work can impose on police officers. Although not limited to mental health crisis calls, the article speaks directly to the broader occupational context in which crisis encounters occur: Sustained exposure to distressing material, moral strain, and emotional labor.

Taken together, the articles in this Research Topic advance an understanding of policing and mental health crises as fundamentally interdisciplinary phenomena. They show that effective crisis response cannot be reduced to single interventions, isolated training programs, or narrowly defined performance metrics. Instead, it emerges from the interaction of individual competencies, organizational cultures, systemic structures, and cross-sector collaboration. By integrating conceptual, methodological, and applied perspectives, the Research Topic contributes to a growing body of scholarship that seeks not only to critique existing practices but also to provide constructive pathways forward.

In framing these contributions, this Editorial also points toward future directions for research and practice. Continued progress will depend on longitudinal evaluations of interdisciplinary models, deeper engagement with lived experience perspectives, and closer alignment between research, policy, and frontline realities. As jurisdictions experiment with new response configurations, the need for theoretically grounded, methodologically rigorous, and practically relevant research becomes ever more pressing. We hope that this Research Topic offers a substantive step in that direction.
